# Experimental study on soil improvement by electrochemical injection of calcium chloride solutions with time interval

**DOI:** 10.1038/s41598-024-66508-w

**Published:** 2024-07-08

**Authors:** Shaoyang Han, Baotian Wang, Yu Wang, Wenpeng Liu, Chenghao Chen, Yijiang Zhang

**Affiliations:** 1 College of Civil Engineering, Jiangsu Open University, Nanjing, 210036 Jiangsu China; 2 Key Laboratory of Ministry of Education for Geomechanics and Embankment Engineering, Hohai University, Nanjing, 210098 Jiangsu China; 3 China Harbour Engineering Company Ltd., Beijing, 100027 China; 4 Department of Geotechnical Engineering, Nanjing Hydraulic Research Institute, Nanjing, 210024 Jiangsu China

**Keywords:** Soil improvement, Electrochemical treatment, Time interval, Calcium chloride, Treatment region, Civil engineering, Electrical and electronic engineering

## Abstract

An improved electroosmotic method is proposed in this paper to enhance the non-uniform effect and efficiency of electroosmotic process. Such method is electroosmotic flow with injection of calcium chloride through the anode, followed by injection through the central tube (a tube at the midpoint between the anode and the cathode) with a suitable time interval between injections. Experimental results indicate that using this method can significantly improve the non-uniform reduction in water content throughout the soil, mitigate the formation of cracks in the anode section, and therefore considerably inhibit the increase in the electric resistance. After treatment, the drained water could be raised to 3.59 times more than that of pure electroosmotic flow, and 1.3 times that of simultaneous injection through both the anode and the central tube with considerably slight increase in power consumption. Moreover, the area of cementation was also expanded, approximately twice larger than that of pure electroosmotic flow and one and a half that of simultaneous injection. It is also worth noting that the proposed method performs better with the same power consumption. The results demonstrate that electroosmotic flow with a suitable time interval between injections could improve the efficiency of electroosmotic process and expand the treatment region in soils, hence can be a promising and economic technique for soil improvement in practical engineering.

## Introduction

Electroosmotic consolidation is a ground improvement method for soft soils in which pore water is driven from the anode to the cathode under the electric field to realize consolidation of soils^1,2^. The pioneering work in enhancing characteristics of soils through electroosmotic techniques was first introduced by Casagrande (1949)^3^. Since then, a variety of successful cases and studies have been conducted to further investigate the effects of electroosmotic treatments^4,5^.

In recent years, the method of injecting chemical solutions into soils during electroosmotic flow has been widely adopted to enhance the effect of electroosmotic process^6,7^. This improvement of soil induced by electroosmotic flow with chemical injection is achieved as a result of two processes: the drainage of pore water and a series of chemical reactions^8^. Various solutions have been selected as the injection compound during electroosmotic flow, such as CaCl_2_^9^, Ca(NO_3_)_2_, NaOH and Na_2_SiO_3_^10^, methacrylate polycations^11^, Al_2_(SO_4_)_3_^12^, Mg(CH_3_COO)_2_, AgNO_3_ and ZnSO_4_^13^. These electroosmotic chemical methods are expected to increase the soil conductivity and hydration of cation, resulting in more absorbed water migrating towards the cathode. Furthermore, the injection of chemical solutions will increase the ion exchange effect and cause the flocculation and coagulation of soil particles, thereby greatly improving the electroosmotic consolidation performance^14,15^. However, the treatment area is mostly limited to the anode or cathode region^16,17^. This is due to the formation of acidic and alkaline conditions around the anode and cathode, respectively, under which the chemical reactions between solutions and soil would preferably occurs to produce cementing precipitations^17^.

To overcome the challenge of inhomogeneous effect in electroosmotic consolidation, Chien et al.^17^ introduced a novel technique by installing a relay pipe between the anode and cathode to expand the region of electroosmotic improvement. Further, a method involving electrode polarity reversal^18^ was adopted to make soil strength homogeneous. However, polarity reversal may result in a significantly increase in electrode interface resistance, leading to low current and inefficient energy^19,20^. Moreover, electroosmotic flow together with vacuum preloading was also used to significantly and uniformly improve soil strength^21^, but the practical application of vacuum preloading combined with electroosmotic flow is extremely difficult, because the membrane used in vacuum preloading cannot maintain the tightness of the seal under the electric field conditions.

To further expand the region of improvement and enhance the efficiency of electroosmotic process, an improved method of a tube, which was installed between the anode and the cathode, was developed for the electroosmotic flow with injection of Calcium chloride (CaCl_2_) through the anode, followed by injection through the central tube with an appropriate time interval between injections was studied. For comparison, the electroosmotic process with different injection methods of chemical solutions were also studied. CaCl_2_ was chosen as the injection solution due to advantages, including non-toxicity, non-contamination, and low cost. Treatment effect and further understanding of mechanism were also investigated by monitoring the drained water, drainage rate, electric current, power consumption, settlement, electroosmotic permeability, water content and penetration resistance of soil.

## Experimental study

### Experimental apparatus

**Figure 1:**
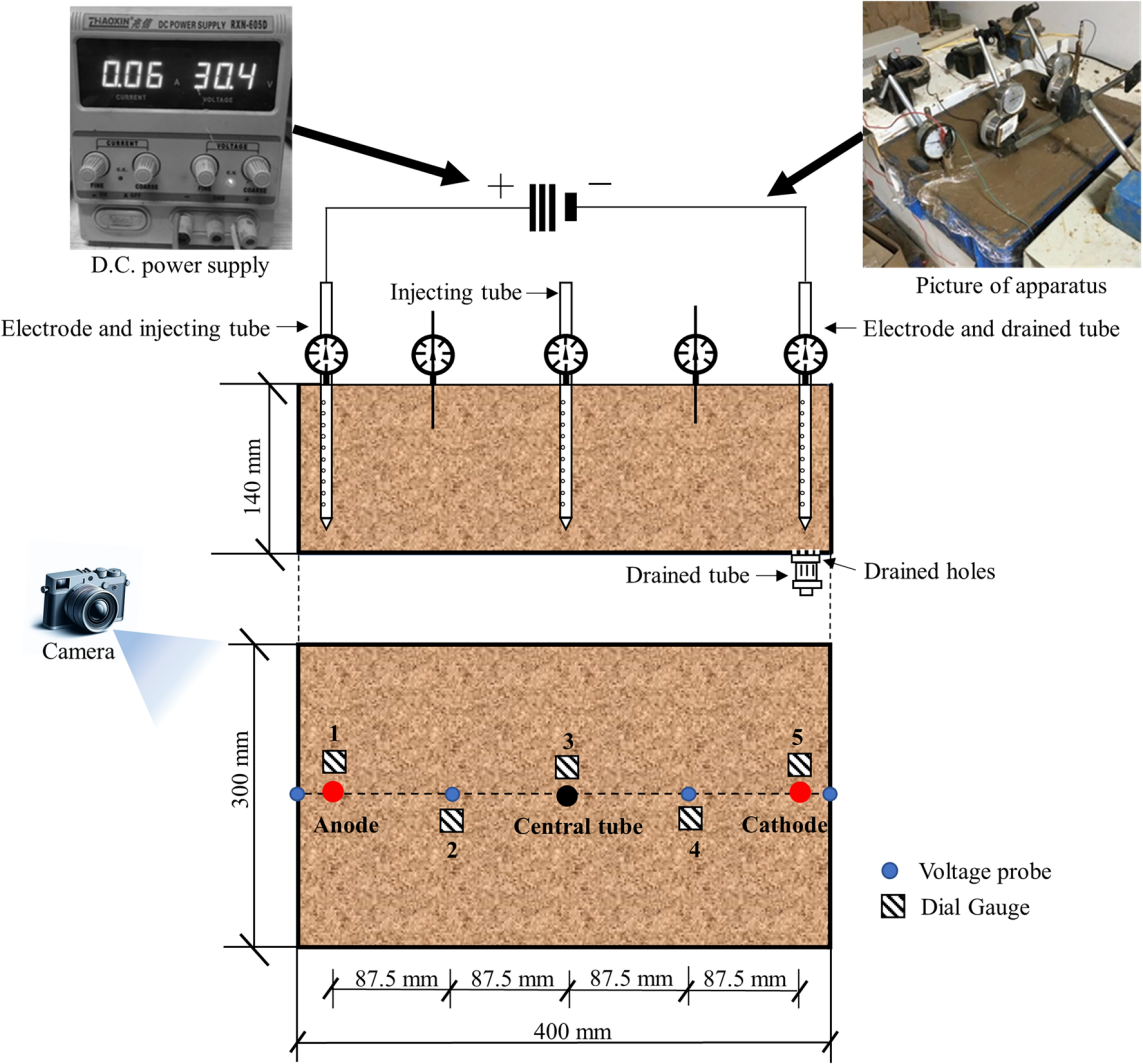
Schematic configuration of test apparatus.

Figure 1 displays the details of the apparatus used in this study. The experiment apparatus consists of an electrokinetic cell and a D.C. power supply device. The electrokinetic cell is made of acrylics with dimensions of 440 mm in length, 330 mm in width 140 mm in height and 10 mm in thickness. Similar to the experiments performed by Chien et al.^17^, tubular stainless steel tubes were used as electrodes and the central tube, with the holes drilled along the surface of the tube to inject (anode and central tube) and drain (cathode) during the electroosmotic process. These two electrodes were 350 mm apart and were both connected to a D.C. power supply device. The D.C. power supply device has an output voltage of up to 60 V and a current of 5 A. A number of holes with a diameter of 3 mm were installed at the bottom of the cell at a distance of 10 mm from the cathode for drainage. The drainage could be controlled through drained tube at the end of the cell during test.

Five voltage probes were installed on top of the cell to measure the voltage in certain time intervals during the test. Five dial gauges were also mounted on the top plater to measure the vertical deformation caused by consolidation. The detailed positions of voltage probes and dial gauges are also shown in Fig. 1. The drained water volume and voltage were measured using a measuring cylinder and a multimeter, respectively. In addition, a camera was applied during the experiments to investigate the behavior of soil-anode interface.

### Materials

The soil used in this study was sampled from Jiangning District, Nanjing, China. The physical properties of the soil were determined according to Chinese Standard GB/T 50123-2019 (Standard for soil test method)^22^ and are summarized in Table 1. The soil can be classified as the clay with low plasticity (CL) according to the Unified Soil Classification System (USCS)^23^. The calcium chloride (CaCl_2_) solution was used as the injection compound during the electroosmotic process.

**Table 1:** Physical properties of the soil.

Specific gravity (Gs)	Liquid limit (LL)	Plastic limit (PL)	Plastic index (PI)	USCS classification
2.67	41.8	23.6	18.2	CL

### Soil samples

A specific amount of air-dried soil was firstly thoroughly mixed with distilled deionized water by a mechanical mixer to achieve a water content of 1.5 times greater than the liquid limit. The mixture was kept in an airtight container with sealed lid for 3 days for moisture equilibration. Then the prepared soil sample was placed in the electrokinetic cell by 5 layers (the holes for the electrodes and central tube were reserved), and a saturated geomembrane was covered on top of the soil to create a horizontal flow condition.

### Test procedure

**Figure 2:**
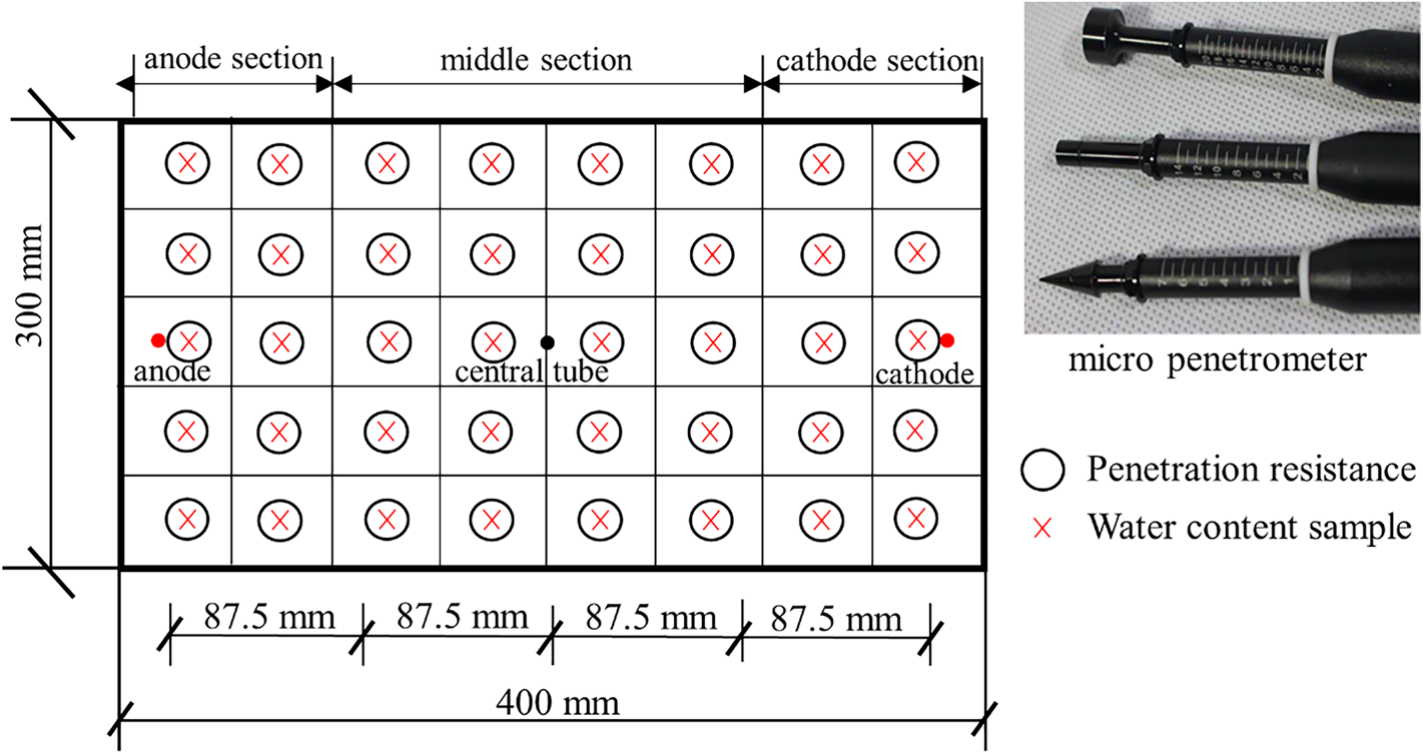
Plan view of laboratory penetration resistance tests and water content collection locations.

A total of four electroosmotic tests were carried out, consisting of one pure electroosmotic test without injection and three tests of electroosmotic with injection of CaCl_2_. A direct current with the voltage of 30 V and the voltage gradients of 50 V/m was applied to the soil to undertake electroosmotic process. The treatment time was 25 h for each test. Two different injection volumes of CaCl_2_ solution, i.e., 70 and 140 mL, with a concentration of 2 mol/L, were injected from the anode or the central tube, and water was discharged from the cathode during electroosmotic process. The voltage, current, surface settlement and drained water from the cathode were monitored during the test. The penetrometer resistance of the soil sample was measured using a specially designed laboratory micro penetrometer (Fig. 2). The micro penetrometer consists of 3 dynamometers (I, II and III) and 3 probes (A, B and C). After the test, the penetrometer resistance values and water content were determined at various locations throughout the sample, as displayed in Fig. 2. The digital data logger took the readings electronically. The procedures for the four different tests are described below.

Four electroosmotic tests are denoted as S1, S2, S3 and S4 respectively. As presented in Table 2, S1 refers to pure electroosmotic flow, S2 refers to electroosmotic flow with injection through the anode only, S3 represents electroosmotic flow with simultaneous injection through both the anode and central tube, and S4 represents electroosmotic flow with injection through the anode followed by injection through the central tube with a 12.5-h interval between injections. In general, the procedure of S1 was enforced by applying a D.C. current through the soil matrix without injection for 25 h, while the electroosmotic flow tests with chemical injection are further categorized. To be specific, for test S2, 140 mL of CaCl_2_ solution was injected into the anode immediately after powering on, and the electroosmotic process will continue for the whole treatment time of 25 h. For test S3, 70 mL of CaCl_2_ solution was firstly injected into the anode and central tube simultaneously immediately after powering on, and the electroosmotic process will continue for the whole treatment time of 25 h. While for test S4, the procedure includes two steps, a first step with CaCl_2_ solution injecting into the anode followed by a second step with CaCl_2_ solution injecting into central tube during electroosmotic process. Specifically, 70 mL of CaCl_2_ solution was firstly injected into the anode immediately after powering on, then the electroosmotic process will last for 12.5 h. After that, another 70 mL of CaCl_2_ solution was injected into the central tube, and the electroosmotic process will last for another 12.5 h. A detailed schematic diagram of these four tests are tabulated in Table 2.

**Table 2:** Schematic diagram of the tests.

Test number	Schematic diagram
S1	
S2	
S3	
S4	

## Results

### Drained water, drainage rate and electric current

**Figure 3:**
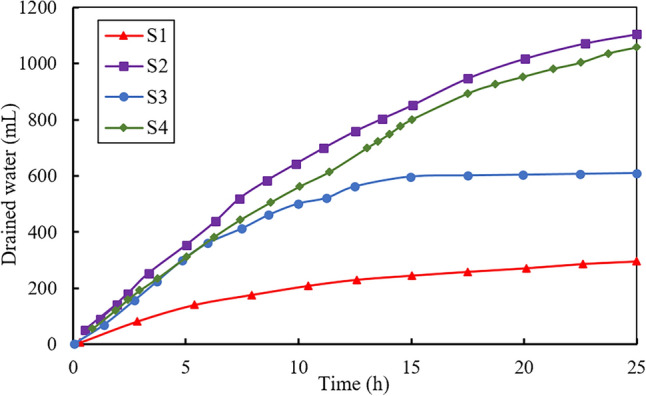
Drained water versus time during tests.

Figure 3 displays the drained water (mL) vs. time (h) during the electroosmotic process for the four tests. The total volume of the drained water was 295, 1107, 607 and 1058 mL for tests S1, S2, S3 and S4, respectively, indicating that electroosmotic flow with injection of CaCl_2_ through the anode accounted for the best drainage performance while the pure electroosmotic flow without chemical injection accounted for the worst. It is worth noting that for the same treatment condition (through both the anode and central tube), the amount of drained water of S4 is 1.74 times more than that of S3, implying that the time interval between two injections through the anode and central tube has a significant impact on the drained water.

**Figure 4:**
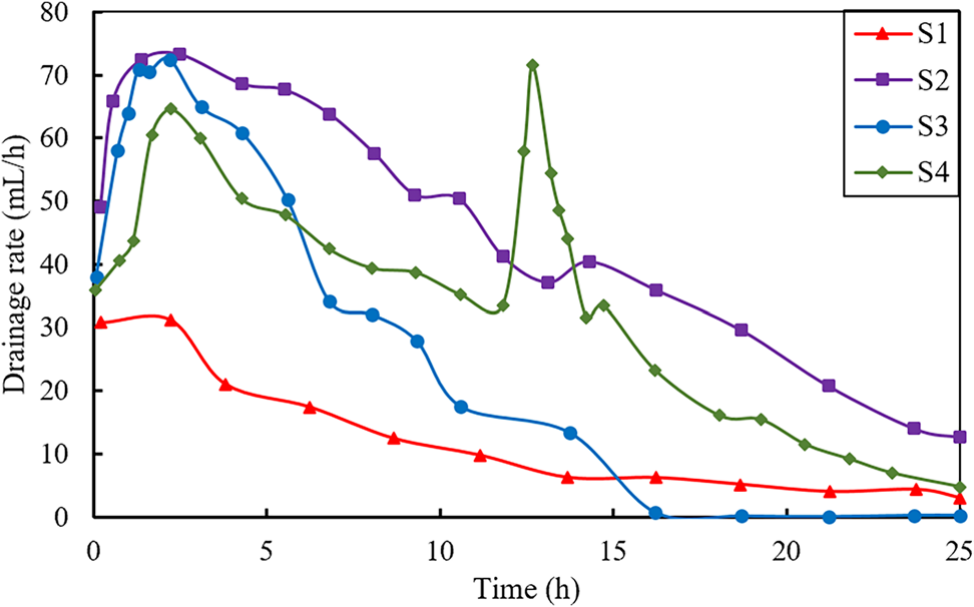
Drainage rate versus time during tests.

Figure 4 displays the drainage rate (mL/h) vs. time (h) during the electroosmotic process for the four tests. As shown in Fig. 4, the initial drainage rate of soil samples ranged from 30 to 40 mL/h, where S1 shows the lowest value of 30.8 mL/h. The drainage rate of S1 gradually decreases over time until reaching a stable state after approximately 15 h, with final drainage rate value about 2.9 mL/h. The drainage rate vs. time curves for tests S2 and S3 show similar variation tendency. For both S2 and S3, the drainage rate initially increases rapidly and reaches a peak at 2.25 h, with values of 73.3 and 72.5 mL/h, respectively. This is followed by a slow decrease in the next 13 to 15 h and afterwards a stable stage. In contrast, the drainage rate vs. time curve for S4 displays a bimodal drainage behavior. This can be attributed to the reinjection of CaCl_2_ solution into the central tube in the middle of the electroosmotic process. The water drainage rate of S4 is similar to those of S2 and S3 in the first 2.25 h, showing a rapidly increasing stage where the initial drainage rate is increase from 35 to 64.6 mL/h. The peak value of S4 at 2.25 h is smaller than those of S2 and S3, which is primarily due to the difference in injection methodology compared with S2 and S3. Comparing S4 with S2, the volume of CaCl_2_ solution at the initial stage of S4 is only half that of S2 through the anode, as a consequence, the peak value of S4 at 2.2 h is 64.6 mL/h which is lower than that of S2. Besides, CaCl_2_ solution was injected from both the anode and central tube at the initial stage for S3, causing simultaneous drainage in both anode and middle section of the soil matrix and leading to a relatively higher peak value compared to S4. Afterwards, the second peak of S4 curve appears around 12.75 h, with a remarkable drainage rate of 72.5 mL/h. This is due to the introduction of additional CaCl_2_ solution into the central tube, which forms new drainage zones and consequently results in a rapid drainage phase.

**Figure 5:**
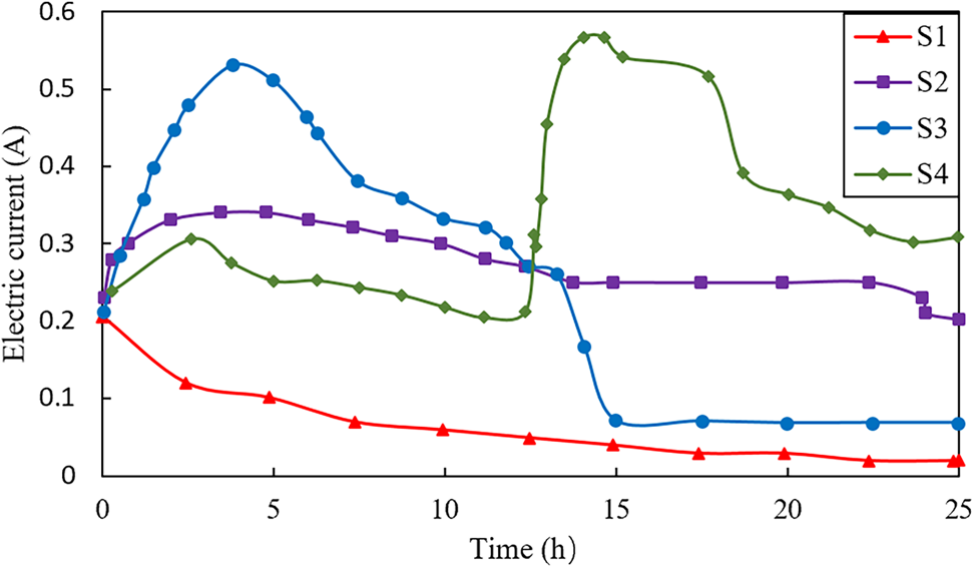
Electric current rate versus time during tests.

Figure 5 shows the electric current (A) through the sample vs. time (h) during the electroosmotic process for the four tests. During electroosmotic process, the electric current of S1 exhibits a continuous decrease until reaching a state of stability. The change in electric current of S2 and S3 tests are similar in the first 5 h, showing a rapid increase to more than 0.3 A due to the presence of salt solution, as well as the desorption and mobilization of ions in the soil matrix^24^. After that, the change in electric current is similar to that in drainage rate, presenting a decreasing stage where the value of electric current decreases from 0.34 A for S2 and 0.53 A for S3 to 0.20 A and 0.06 A, respectively. This reduction suggests a diminished electro-osmotic efficiency, potentially due to a decrease in the gradient of ionic concentration and a partial saturation of charge sites within the clay^25^. Comparing with abovementioned three tests (S1, S2 and S3), the current–time curve of S4 displays a bimodal fluctuation. The electric current for S4 initially increases and reaches the first peak current value of 0.3 A at approximately 2.5 h. This is followed by a slightly decreasing stage and then a secondary increase once power is resumed, which is much higher than the magnitude of the first peak value. The first rise in current may be attributed to the enhanced ionic mobility induced by the introduction of CaCl_2_ to the anode. The subsequent reduction could be indicative of a temporary electrochemical equilibrium being reached within the clay matrix. The introduction of additional CaCl_2_ solution into the central tube leads to a significant increase in the total ion concentration within the soil matrix, resulting in a decrease of resistance and an increase of current flow in the vicinity of the central grouting tube. In general, staged injection could create zones of enhanced electroosmotic activity at different times, contributing to the sustained drainage over the duration of the experiment.

### Power consumption

The economics of electroosmotic chemical treatment method are governed by the power consumption. The electrical power consumption, $W$, can be calculated using:

1 $$W = \int {\frac{V \cdot I \cdot t}{{1000}}} dt$$

where $V$ is the applied vlotage (V), $I$ is the electric current as a fuction of time (A), and $t$ is the processing time (h).

**Figure 6:**
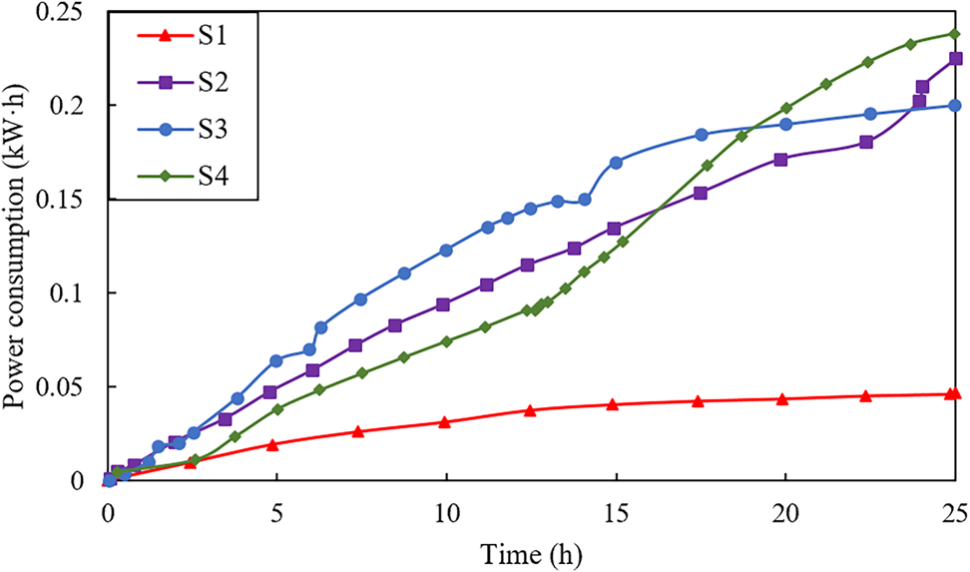
Power consumption versus time during tests.

Figure 6 shows the power consumption (kW h) vs. time (h) during the electroosmotic procss for the four tests. The power consumption after treatment is also presented in Table 3. As shown, the power consmuption increases with time. The final power consumpion is 0.05, 0.22, 0.20 and 0.24 kW h for tests S1, S2, S3 and S4, respectively. It can be clearly seen that the power consumed by electroosmotic flow with injection is approximately twice that of the electroosmotic flow without injection. This is mainly because the electroosmotic flow with injection will result in an increase in electric conductivity, thus the power required for the process increases proportionally^26^. It is also worth noting that the power consumed in test S4 is slightly greater than that in S2, but the increment is acceptable in the case of the tested soils.

**Table 3:** Summary of power consumption after treatment.

Test number	S1	S2	S3	S4
Power consumption (kW h)	0.05	0.22	0.20	0.24

**Figure 7:**
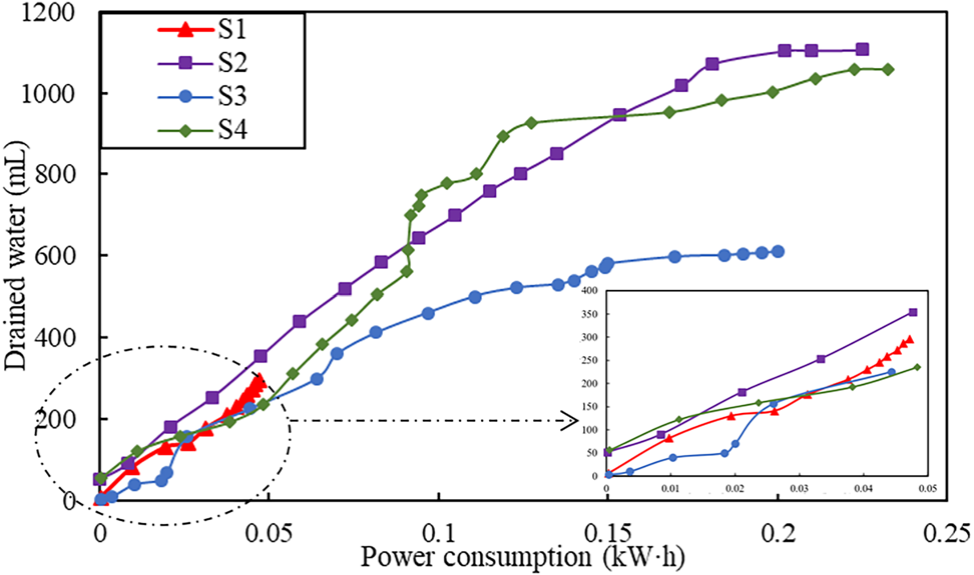
Relationship between drained water and power consumption.

Figure 7 shows the relationship between the drained water and the power consumption. It can be seen that test S1 displays the lowest power consumption but the worst drainage performance. Under the same value of power consumption, the volume of drained water in tests S2 and S4 is significantly greater than that of S3, indicating the higher efficiency in S2 and S4 in this study.

### Settlement

**Figure 8:**
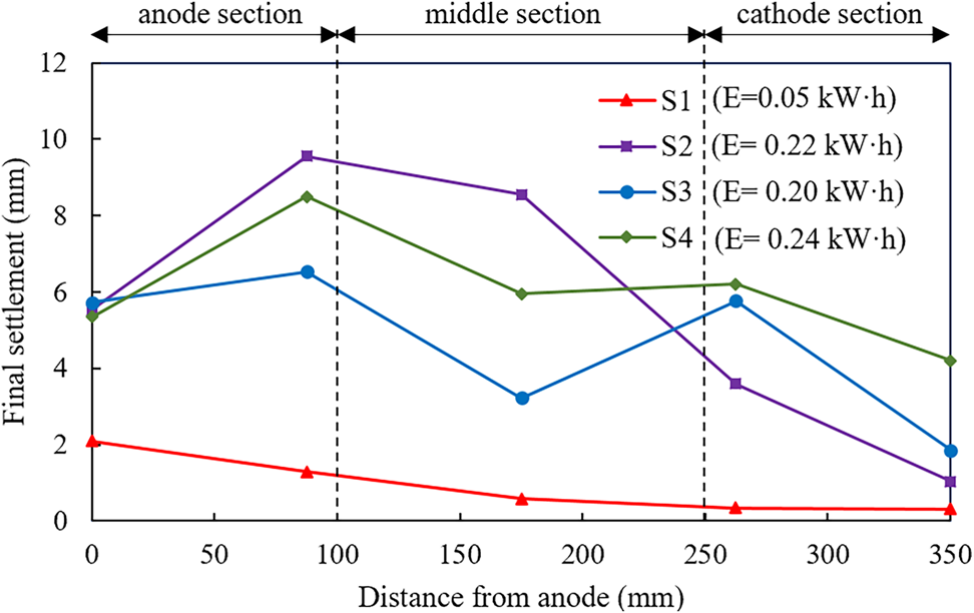
Settlement distribution after treatment.

The settlement distribution after treating vs. the distance from the anode for four different modes are presented in Fig. 8. As shown, the final settlement was measured at five locations, namely 0, 8.75, 17.5, 26.3 and 35 cm away from the anode. It can be readily seen that all the settlements of five locations treated by test S1 keep the lowest, which is consistent with the previously observed lowest drained water among the four tests as shown in Fig. 3. The settlement induced by tests S2, S3 and S4 is around 5.5 cm near the anode, more than twice than that induced by S1, indicating that injection has obviously positive effect on settlement. With the increase of the distance from the anode, the settlement induced by S2 first increases to the largest value 9.6 cm for the measure point 9 cm away from the anode, and then smoothly decreases to 8.2 cm for the measure point 17.5 cm away from the anode. After that, the settlement induced by S2 rapidly decreases to 2 cm for the area near the cathode, which is almost the same with that induced by S1 at the same location. That is to say, although concentrated injection of CaCl_2_ at the anode could largely improve the settlement around the anode, it shows obvious limitation in increasing the settlement near the cathode, which causes highly non-uniform treatment region of the soil matrix. This is because concentrated injection of CaCl_2_ at the anode creates a strong electroosmotic effect nearby, but this effect diminishes with distance as the ionic concentration gradient falls off, leading to less effective consolidation further away^25^.

In contrast, a uniform improvement of S3 and S4 was observed at various locations throughout the sample, suggesting that the injection of CaCl_2_ through both anode and central tube could effectively expand the region of treatment. Furthermore, it can be noted that S4 produces a larger settlement magnitude compared with S3. This is mainly because appropriately prolonging second injection interval can significantly re-establish the electrochemical gradient after the initial injection. This gradient is further utilized during the secondary injection, leading to increased water movement and consequently resulting in an enhancement in the final settlement, a more detailed explanation will be provided in the Discussion section. Therefore, test S4 can significantly reduce the non-uniform electrochemical changes in the treated samples, such that producing the relatively uniform and considerable settlement throughout the sample.

### Electroosmotic permeability

**Figure 9:**
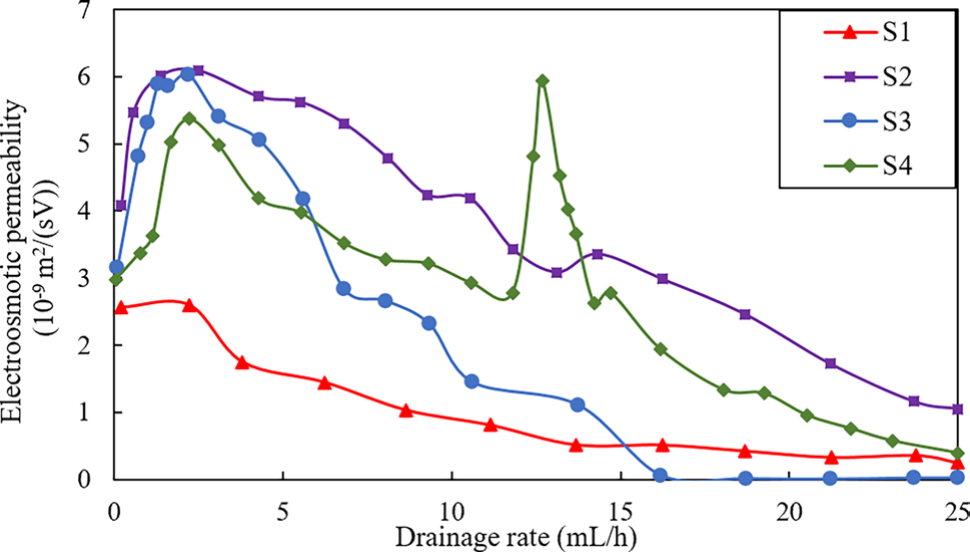
Relationship between electroosmotic permeability and drainage rate.

The electroosmotic permeability ($k_{{\text{e}}}$) governs the electroosmotic flow in a soil mass, hence can be used to estimate the effectiveness of electroosmotic process in soils^27^. $k_{{\text{e}}}$ is defined by an empirical relation

2 $$k_{e} = \frac{Q}{E \times A}$$

where $Q$ is the electroosmotic flow drainage rate, $A$ is the cross-sectional area normal to the direction of flow and $E$ is the electrical field intensity. Based on Eq. (2), the values of $k_{{\text{e}}}$ for the tests in this study were calculated and the results were presented in Fig. 9. It can be seen that the changes in $k_{{\text{e}}}$ are similar to that in drainage rate, with a rapid stage in the first 2.5 h where the initial $k_{{\text{e}}}$ were 2.60 × 10^−9^, 4.08 × 10^−9^, 3.16 × 10^−9^ and 2.98 × 10^−9^ m^2^/(sV) for tests S1, S2, S3 and S4, respectively. Thereafter a gradually decrease stage followed in which $k_{{\text{e}}}$ finally decreased to 0.24 × 10^−9^, 1.05 × 10^−9^, 0 and 0.39 × 10^−9^ m^2^/(sV). It is obvious that $k_{{\text{e}}}$ of tests S2 and S4 always maintain a relatively high level during the electroosmotic process.

The Helmholtz–Smoluchowski model (H–S model) is commonly used to explain electroosmotic process in soils^28^. In H–S model, $k_{{\text{e}}}$ is denoted by:

3 $$k_{{\text{e}}} = \frac{{n\varepsilon_{{\text{w}}} }}{\mu }\zeta$$

where $n$ is the porosity of soil, $\varepsilon_{{\text{w}}}$ is the permittivity of pore water, $\mu$ is the pore water viscosity, and $\zeta$ is the zeta potential. H–S model suggests that $k_{{\text{e}}}$ is proportional to the zeta potential. The variation of zeta potential is a function of pH changes. As the pH values are not uniform across the sample, the zeta potential is not constant therefore^29^. Zhou et al.^30^ also indicates that the zeta potential decreases with the increase of salinity, which leads to a decrease in $k_{{\text{e}}}$ as suggested by the H–S model (Eq. 3). Since the drained water from the cathode is reduced due to the decrease in $k_{{\text{e}}}$, consolidation settlement is occurring caused by the drained water. Therefore, a higher $k_{{\text{e}}}$ indicates more effective electroosmotic drainage in the soil.

As can be seen from Fig. 9, $k_{{\text{e}}}$ of test S2 is consistently the highest. However, combining the results form Fig. 4 (Drainage rate versus time during tests) and Fig. 8 (Settlement distribution after treatment), it is clear that the uniformity after treatment for test S2 is the worst though the drainage efficiency is the highest. In comparison, despite that $k_{{\text{e}}}$ of the S4 test is slightly lower than that of S2, it is still considerable compared with tests S1 and S3. Combining the settlement results from Fig. 8, it is evident that the S4 scheme could significantly improve efficiency of electroosmotic and expand the treatment region in soil, thus making it superior to other schemes.

### Water content and penetration resistance

**Figure 10:**
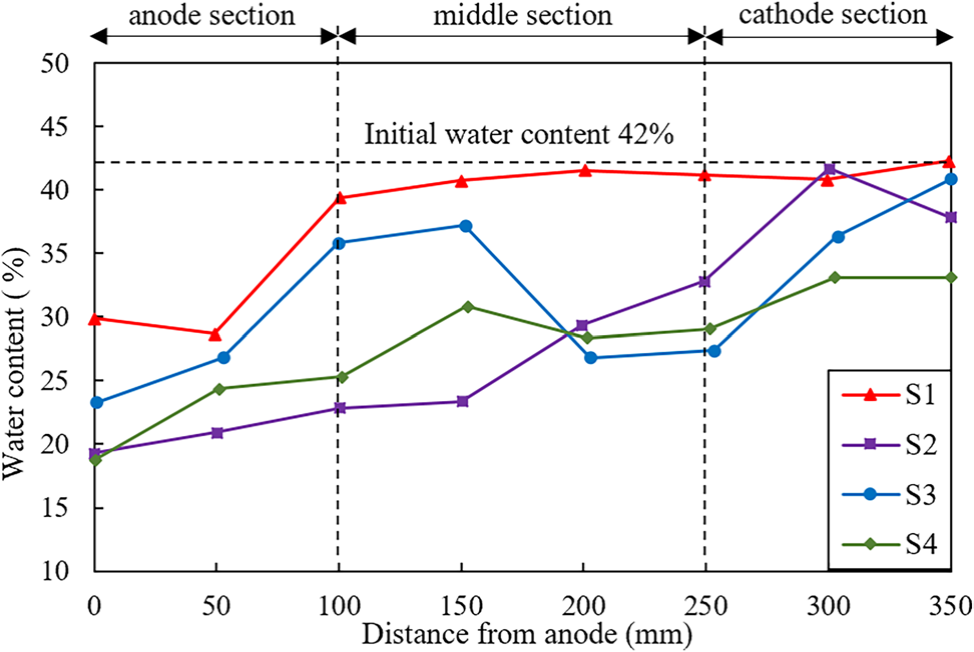
Water content distribution after treatment.

The water content after treatment vs. the distance from the anode for four tests are presented in Fig. 10. The initial water content of soil samples for all the four tests is 42%. It can be observed that the distribution of water content is highly non-uniform for S1 after treatment. A reduction in water content from 42 to 28% is noticed surrounding the anode in S1, while the water content of the remaining regions over the entire sample exhibits rarely changes compared with the initial water content of 42%. Similarly, the distribution of water content over the sample is also non-uniform in S2, with the water content near the anode being almost half of that near the cathode. For S3, a significant reduction in water content is observed in the proximity of the anode and central tube, while the reduction range gradually decreases with the increase of distance from these two locations. Note that the decrement in water content near the anode is around 24% for S2 while around 18% for S3. This may result from the injection of a greater volume of CaCl_2_ solution through the anode for S2. In contrast, test S4 exhibits a comparatively gradual decline in water content, indicating a more uniform distribution of moisture throughout the sample after treatment. Specifically, the water content near the anode shows a reduction of 23%, while a decrease of 10% is observed near the cathode. In general, test S4 can significantly improve the non-uniform reduction in water content of the treated samples, while the decrement in water content is remarkable compared with other three tests.

**Figure 11:**
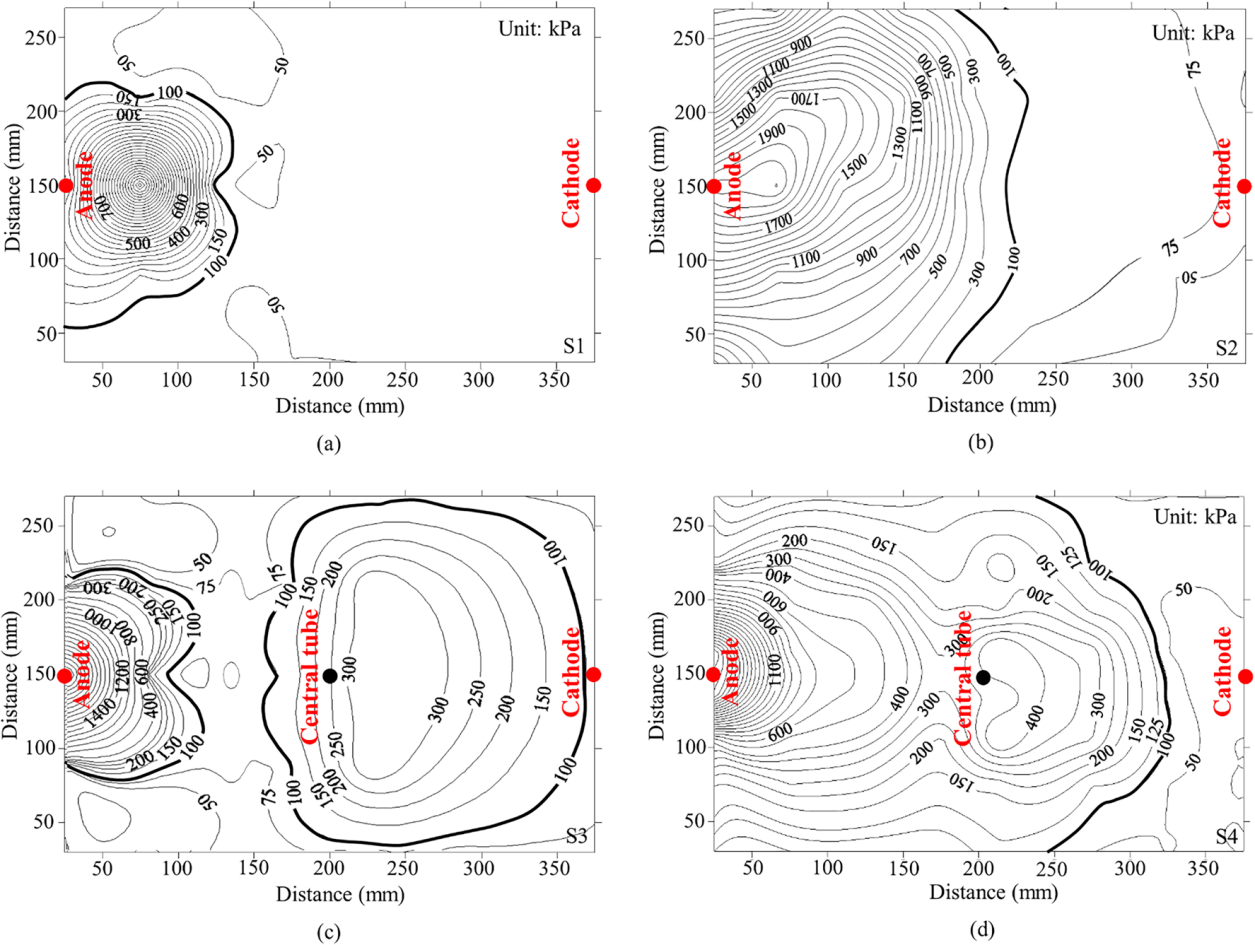
Penetration resistance contour plot after treatment (**a**) S1 (pure electroosmotic flow) (**b**) S2 (electroosmotic flow with injection through the anode) (**c**) S3 (electroosmotic flow with simultaneous injection through both the anode and central tube) and (**d**) S4 (electroosmotic flow with injection through the anode followed by injection through the central tube with a 12.5-h interval between injections).

Figure 11 shows the contour plots of penetration resistance for the entire sample after treatment for four tests. The contour plots were generated using Kriging method^31^, which is a spatial interpolation estimator that is applied to find the best linear unbiased estimate at each location and is determined according to the linear combination of the known values of all sampled locations. The initial penetration resistance ranged from 50 to 75 kPa, with an average water content of 42%. For S1, the contour lines representing the distribution of penetration resistance are densely packed surrounding the anode, while the majority of the remaining soil area exhibits no significant growth in penetration resistance and maintains a constant resistance of 50 kPa. In contrast, the contour lines of S2 are more dispersed compared with those of S1. However, S2 also exhibits a non-uniform strength increment over the whole sample, with a relatively high penetration resistance value of 1900 kPa near the anode and quite a low value of 50 kPa near the cathode. Furthermore, it is apparent that the soils, not only between the anode and the cathode, but also away from the alignment of the anode and the cathode, are significantly improved in S3 and S4. The penetration resistance of S3 and S4 is about 1400 and 1100 kPa surrounding the anode, about 300 and 400 kPa surrounding the central tube, and about 100 and 75 kPa surrounding the cathode, respectively.

**Figure 12:**
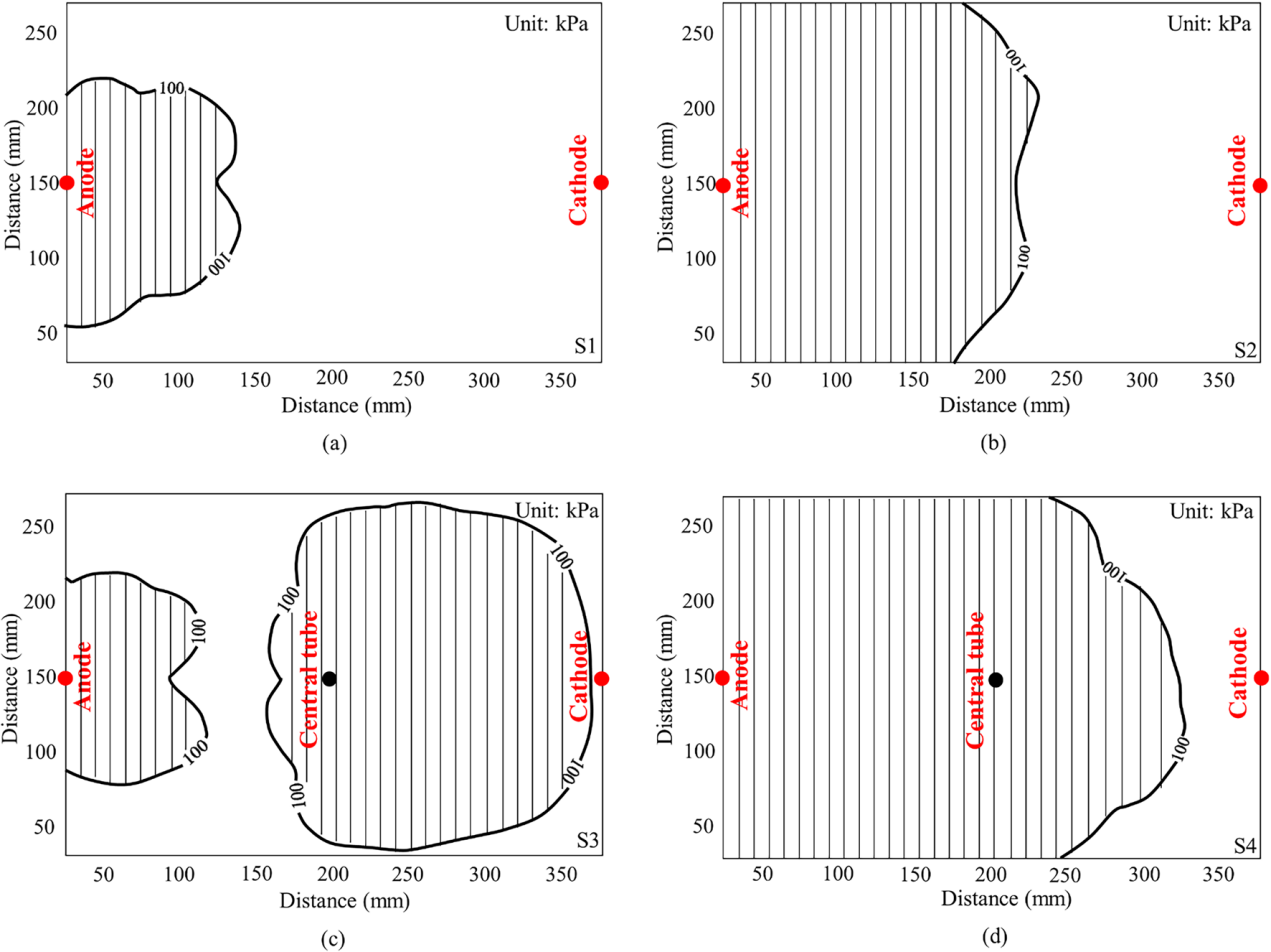
Comparison of the cementation area after treatment (**a**) S1 (pure electroosmotic flow) (**b**) S2 (electroosmotic flow with injection through the anode) (**c**) S3 (electroosmotic flow with simultaneous injection through both the anode and central tube) and (**d**) S4 (electroosmotic flow with injection through the anode followed by injection through the central tube with a 12.5-h interval between injections).

It is expected that cementation between soil particles due to the chemical reaction between injected solutions should contribute to a large proportion of the cone resistance when it is greater than 100 kPa. Therefore, for convenience of description, the area of penetration resistance greater than 100 kPa after treatment is defined as a cementation area, as shown in Fig. 12, similar to the method used by Chien et al.^17^. It is calculated from Fig. 12a and b that the cementation area for S1 and S2 are about 18.05 and 52.98% of the entire sample, respectively. It is also evident that the cementation area in S3 is larger than that in S1 and S2, accounting for about 62.30% of the total area (Fig. 12c). However, the cementation area of S3 remains limited to the anode and the central tube region, the treatment effect is still very weak outside the regions. Figure 12d shows a uniform improvement region throughout the sample, with a cementation area accounting for 80.6% of the entire sample for S4. This observation indicates a highly heterogeneous distribution of the treatment area of S4. Thus, it can be concluded that prolonging the interval between injections can remarkably cause an increase in cementation area.

## Discussion

It is well known that the primary mechanism of the electroosmotic improvement without injection of solutions is the attraction of cations towards the cathode^1,32^. As a result, water moves towards the cathode along with the cations, driven also by the electric potential.

As for electroosmotic flow with injection, in addition to the pure electroosmotic effects, the increase of cation in the soil induced by the injection of saline solutions will result in an increase in electric conductivity and hydration of cation, which causes more absorbed water, along with the cation, to migrate towards the cathode. The water content thus decreases with the increase of distance from the anode. The electroosmotic effect is certainly enhanced, compared with the electroosmotic flow without injection^33^. Furthermore, the exchange of ions on the surfaces of soil particles will lead to flocculation and coagulation of soil particles, resulting in larger colloids and an overall increase in soil strength. Although electroosmotic flow with injection through the anode is effective in strengthening soil, the treated soil is, however, obviously limited to the anode region^4,17^.

As stated, it is confirmed by the fact that injection of CaCl_2_ through both the anode and central tube could effectively expand the treatment region. This may be primarily due to the following two reasons: first, water can be drained simultaneously in both anode and middle section of the soil, thereby expanding the consolidation area, and second, the formation of reaction products and precipitations were accumulated and thus clogged the pore spaces near both the anode and middle section of soil matrix, and therefore the area of cementation would be expanded. However, this clogging effect would also hinder the flow of water and reduce the overall permeability of the soil, resulting in a progressive decrease in drainage efficiency^32,34^. In addition to the clogging effect mentioned above, the electric resistance of the entire sample of S3 and S4 should be further discussed, as the efficiency of electroosmotic treatment is substantially controlled by the electric resistance of system^1,35^.

**Figure 13:**
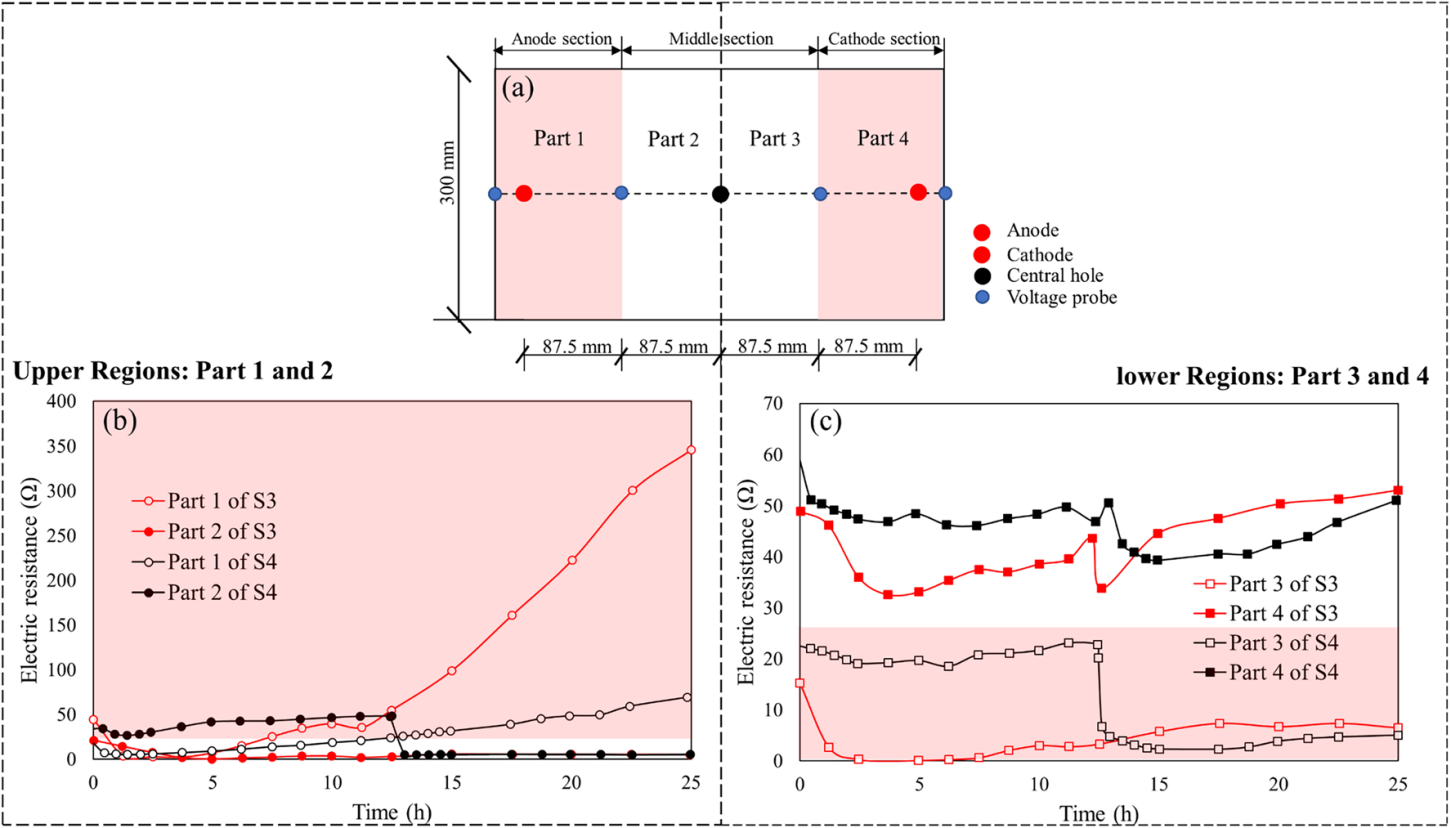
Electric resistance versus time during tests (**a**) Schematic diagram of sample segmentation (**b**) electric resistance of up regions (part 1 and 2) and (**c**) electric resistance of lower regions (part 1 and 2).

**Figure 14:**
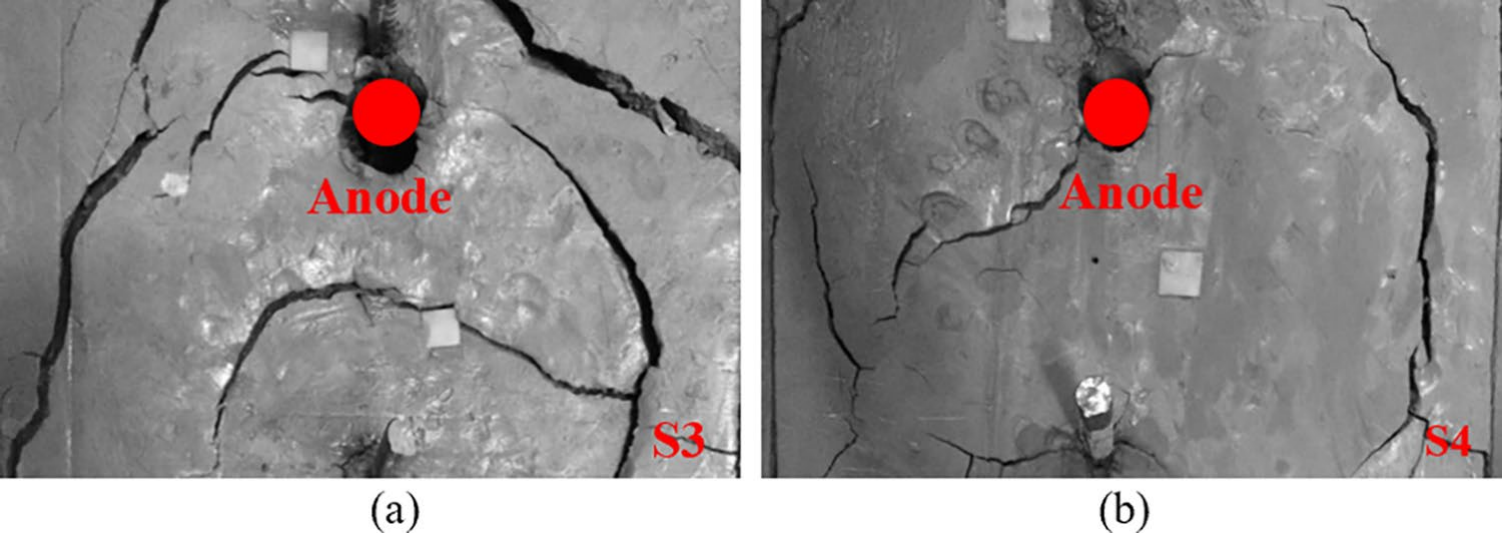
Images of the soil anode interface after treatment (**a**) anode section of S3 and (**b**) anode section of S4.

Figure 13 shows the electric resistance (Ω) vs. time (h) during the electroosmotic process for S3 and S4. According to the electrical current and voltage data, the electric resistance of part 1 (the soil near anode), part 2 (the soil near the left side of the central tube), part 3 (the soil near the right side of the central tube) and part 4 (the soil near cathode) of soil matrix with regard to time can be achieved, as shown in Fig. 13 (a). Generally speaking, the whole soil sample could be separated to two regions by the central axis, i.e. upper regions including part 1 and part 2, and lower regions including part 3 and part 4. In the first period of 12.5 h, it is observed that the electrical resistance in S3 is obviously lower than that in S4. This is because the volume of CaCl_2_ solution injected in S3 is twice that in S4 in the first 12.5 h, and consequently resulting in a higher electric current (as shown in Fig. 5)^33^. After that, in the second period of 12.5 h, a continuous and substantial increase in electric resistance is observed in the anode section (part 1) of S3, reaching a final value of 345 Ω at the end of treatment, which is approximately 5 times higher than that of S4. The occurrence of cracks should account for the sharp increase in electrical resistance near the anode in S3. During electroosmotic process, water close to the anode will be heavier electrolyzed and therefore a volume shrinkage appeared in the soil in the anode section (part 1) (as shown in Fig. 14a). These cracks caused significant voltage loss, which further led to decrease in electric current and drainage rate^24^. It is apparent from Fig. 14b that the injection of CaCl_2_ solution through the anode followed by injection of the CaCl_2_ solution through the central tube after 12.5 h can effectively mitigate the formation of cracks in the anode section, and therefore considerably reduce the increase in the electric resistance. Additionally, appropriately prolonging second CaCl_2_ injection interval through the central tube can re-establish the electrochemical gradient after the first injection, causing an increase in electric current and a decrease in electric resistance. Therefore, to improve the effect of electroosmotic process on soil, the shrinkage of soil should be restrained by methods such as prolonging second CaCl_2_ injection interval through the central tube during electroosmotic flow.

## Conclusions

In this study, the effect of electroosmotic process with and without injection were investigated through laboratory tests. One pure electroosmotic flow test without injection and three tests of electroosmotic flow with injection (electroosmotic flow with injection of CaCl_2_ solution through the anode only, electroosmotic flow with simultaneous injection of CaCl_2_ solution through both the anode and central tube, and electroosmotic flow with injection of CaCl_2_ through the anode followed by injection through the central tube with a 12.5-h interval between injections) were performed to access the effectiveness of the treatment. Drained water, drainage rate, electric current, power consumption, settlement, electroosmotic permeability, water content and penetration resistance were analyzed to investigate drainage and consolidation behaviors. Based on the results of this study, the following conclusions can be drawn:

1. The effect of electroosmotic flow can be improved by the injection of CaCl_2_ solution during electroosmotic process. After electroosmotic flow with injection of CaCl_2_ solution through the anode for a period of 25 h, the drained water of soil was about 3.75 times more than that of pure electroosmotic flow without injection. The cementation area obtained by electroosmotic flow with injection through the anode was increased to 52% of the entire sample, compared to that only 18% obtained by pure electroosmotic flow. However, the results also show that the region of improvement is generally limited to the anode section using electroosmotic flow with injection through the anode only.
2. Electroosmotic flow with simultaneous injection through both the anode and central tube could effectively expand the treatment region, and the cementation area is nearly 3.45 times larger than that of pure electroosmotic flow and 1.17 times that of injection through anode only. Nevertheless, due to the clogging effect and crack appeared in the anode section, the overall permeability of the soil was reduced, resulting in progressively lower drainage efficiency.
3. Electroosmotic flow with injection through the anode followed by injection through the central tube with a 12.5-h interval between injections could improve the cementation area to nearly cover the entire sample, with a cementation area value of 80.6% of the entire sample, and the corresponding drained water could be raised to 3.59 times more than that of pure electroosmotic flow and 1.74 times that of simultaneous injection through both the anode and central tube. In addition, prolonging the second CaCl_2_ injection interval through the central tube can effectively mitigate the formation of cracks in the anode section, which will considerably reduce the increase in electric resistance.
4. The results indicate that electroosmotic flow with injection of CaCl_2_ through the anode followed by injection through the central tube with a suitable interval between injections may be a potential technique for the improvement of soft clay.

## Data Availability

The datasets analyzed during the current study are available from the corresponding author on reasonable request.
